# Targeting type 2 inflammation in dermatology: mechanisms and clinical implications

**DOI:** 10.3389/fimmu.2026.1917593

**Published:** 2026-08-17

**Authors:** Nicole Khalil, Leah Farhadi, Gil Yosipovitch

**Affiliations:** Dr. Phillip Frost Department of Dermatology and Cutaneous Surgery, University of Miami, Miller School of Medicine, Miami, FL, United States

**Keywords:** biologic therapies, chronic pruritus, cytokines, inflammatory dermatoses, neuroimmune crosstalk, type 2 inflammation

## Abstract

Type 2 inflammation is a fundamental immunologic pathway underlying a growing spectrum of dermatologic diseases. While classically associated with atopic dermatitis, type 2 immune responses are increasingly recognized as key contributors to prurigo nodularis, chronic spontaneous urticaria, bullous pemphigoid, lichen simplex chronicus, and other chronic pruritic disorders. Barrier disruption and epithelial injury initiate the release of alarmins, including thymic stromal lymphopoietin (TSLP), interleukin (IL)-33, and IL-25, which activate innate and adaptive immune pathways that promote type 2 inflammation. Subsequent production of effector mediators such as IL-4, IL-13, IL-5, IL-31, immunoglobulin E (IgE), and periostin drives barrier dysfunction, immune cell recruitment, tissue remodeling, and chronic pruritus. Increasing evidence further highlights the importance of neuroimmune crosstalk, with direct interactions between immune cells, cytokines, and sensory neurons contributing to itch sensitization and disease persistence. The clinical success of biologic therapies targeting IL-4/IL-13, IgE, and IL-31 signaling has provided powerful functional validation of these pathogenic pathways and has transformed the management of multiple inflammatory skin diseases. In this review, we summarize the molecular and cellular mechanisms underlying type 2 inflammation in the skin, discuss its role across diverse dermatologic conditions, and highlight emerging concepts in disease heterogeneity, neuroimmune signaling, and precision medicine. Collectively, these findings support type 2 inflammation as a unifying immunologic framework and therapeutically actionable target across dermatology.

## 1 Introduction

Type 2 inflammation has emerged as a central immunologic pathway underlying a growing spectrum of dermatologic diseases. Characterized by the coordinated activity of T helper 2 (Th2) cells, group 2 innate lymphoid cells (ILC2s), and their associated cytokines—including interleukin (IL)-4, IL-13, IL-5, and IL-31 (1, 2)—this immune response was initially recognized for its role in host defense against parasites (3). However, chronic activation of type 2 immune pathways can drive pathologic inflammation, resulting in tissue remodeling, immunoglobulin E (IgE) production, eosinophilic infiltration, and chronic pruritus (CP) (3–5). Recent advances have established type 2 inflammation as a key contributor to several common and burdensome dermatologic disorders, expanding its relevance far beyond the traditional paradigm of atopic dermatitis (AD) (4).

The skin is continuously exposed to environmental and microbial stimuli that disrupt barrier integrity (6) and trigger release of epithelial alarmins, including thymic stromal lymphopoietin (TSLP), IL-33, and IL-25 (7). These signals activate ILC2s, dendritic cells, and Th2 lymphocytes (2, 7), leading to production of IL-4, IL-13, IL-5, and IL-31, which drive eosinophil inflammation, barrier dysfunction, and CP through extensive neuroimmune interactions (4, 8).

Recognition of shared type 2 inflammatory mechanisms has transformed the understanding and treatment of multiple dermatologic conditions. While AD remains the prototypical type 2 skin disease, increasing evidence supports a pathogenic role for type 2 immunity in chronic spontaneous urticaria (CSU), prurigo nodularis (PN) (9), bullous pemphigoid (BP), and other chronic pruritic disorders (3, 4). Importantly, the clinical success of targeted biologic therapies has provided therapeutic validation of these pathways and further reinforced the concept of a common type 2 inflammatory axis across diverse diseases (1, 2).

In this review, we summarize the immunologic mechanisms underlying cutaneous type 2 inflammation and discuss their contribution to major dermatologic disorders, and we further examine how advances in biologic therapies have reshaped disease management and consider emerging evidence for the role of type 2-targeted treatments in additional dermatologic conditions.

A narrative literature review was conducted using PubMed to identify relevant studies published through June 2026. Search terms included combinations of “type 2 inflammation,” “dermatology,” “chronic pruritus,” “atopic dermatitis,” “prurigo nodularis,” “chronic spontaneous urticaria,” “bullous pemphigoid,” and “biologic therapy.” Priority was given to mechanistic studies, pivotal clinical trials, systematic reviews, meta-analyses, and current FDA approvals relevant to type 2 inflammatory skin diseases. Reference lists of selected publications were additionally screened to identify relevant studies not captured in the initial search.

## 2 Core components of type 2 inflammation in skin

### 2.1 Initiation by barrier injury and alarmins

Type 2 skin inflammation begins at the epidermal barrier. Genetic factors (e.g., filaggrin (FLG) loss-of-function mutations), mechanical injury (e.g., scratching), microbial dysbiosis (Staphylococcus aureus, Malassezia), and environmental insults compromise the stratum corneum, increasing transepidermal water loss and allergen penetration (10). The resulting damage to keratinocytes results in their release of epithelial alarmins (TSLP, IL-25, and IL-33) that initiate the type 2 cascade upstream (7).

Among the epithelial-derived alarmins, TSLP is considered a master upstream regulator of type 2 immunity, promoting dendritic cell-mediated Th2 polarization through OX40L signaling while directly activating ILC2s, basophils, mast cells, and eosinophils (7, 11). TSLP also perpetuates barrier dysfunction by inducing keratinocyte production of periostin and IL-33 (7, 11). IL-33 signals through the ST2 receptor on ILC2s, Th2 cells, and mast cells, serving as an amplifier of innate type 2 responses (12). IL-25 further reinforces this inflammatory environment by activating ILC2s and Th2 cells through IL-17RB signaling, thereby promoting production of downstream type 2 cytokines and sustaining chronic inflammation (12).

### 2.2 Cellular mediators

Type 2 inflammation is orchestrated by a network of innate and adaptive immune cells. Th2 cells and ILC2s are major sources of IL-4, IL-13, and IL-31 (1, 6, 13), promoting barrier dysfunction, IgE class switching, eosinophilic inflammation, and itch (1, 14). Eosinophils, mast cells basophils, fibroblasts, and alternative activated (M2) macrophages are increasingly recognized as components of type 2 inflammation and may contribute to fibrosis, IL-31 production, and chronic neuroimmune itch (15–20).

### 2.3 Effector cytokines and mediators

Several cytokines play important roles in type 2 inflammation-driven barrier dysfunction, immune cell recruitment, and pruritus. IL-4 and IL-13 are the central effector cytokines of type 2 immunity. Through shared signaling via IL-4Rα and STAT6, they promote IgE class switching, inflammatory cell recruitment, and epidermal barrier disruption (6, 21–23). IL-5 regulates eosinophil activation and survival (6), whereas IL-31 serves as a major pruritogenic cytokine directly activation sensory neurons (22, 24). Periostin amplifies these pathways by promoting TSLP production and sustaining chronic neuroimmune inflammation (19, 20, 25–27).

### 2.4 Neuroimmune crosstalk

**Figure 1:**
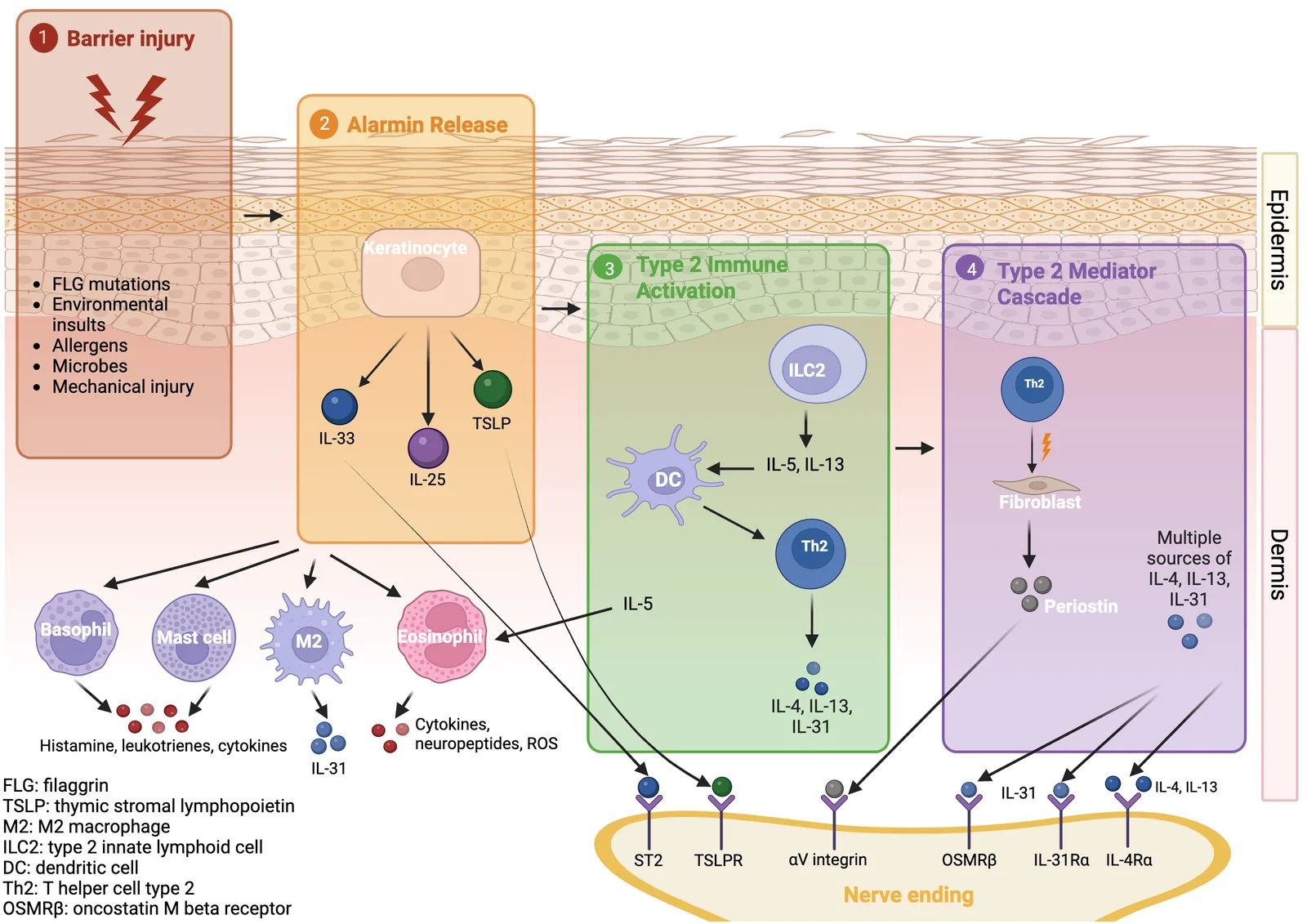
Overview of the pathway and mediators underlying type 2 inflammation in the skin. Figure made using BioRender.com.

Neuroimmune crosstalk is a defining feature of type 2 inflammation and a key driver of CP. Sensory neurons express receptors for multiple type 2 mediators, including IL-4, IL-13, IL-31, TSLP, and periostin, allowing direct communication between the immune and nervous systems. These mediators promote neuronal activation, sensitization, and nerve fiber growth, resulting in heightened itch perception (25, 26). IL-4 and IL-13 binding to IL-4Rα results in JAK signaling that causes acute itch, but these cytokines also act via TRPV1 and TRPA1 to enhance neuronal sensitivity to pruritic stimuli (26). IL-31 also promotes acute itch via interactions with IL-31RA/OSMRβ, however it also plays a role in nerve fiber elongation and sustaining chronic itch (26). TSLP receptor is similarly expressed on a discrete population of pruriceptive sensory neurons, allowing TSLP released from damaged keratinocytes to act directly on nerve fibers rather than solely through ILC2/Th2 intermediates (26, 28). Periostin further sensitizes this circuit by potentiating TSLP release and promoting nerve fiber outgrowth into the epidermis, increasing cutaneous nerve density and lowering the itch threshold (28). This loop lowers the threshold for IL-31 to act on IL-31RA/OSMRβ-expressing sensory neurons, while IL-31 itself promotes further keratinocyte activation and neuronal sprouting (26, 29). Bidirectional interactions between sensory neurons and mast cells further amplify inflammation, as neuronal neuropeptides trigger mast cell degranulation and mast cell-derived mediators activate pruriceptive neurons (30). Together, these self-sustaining neuroimmune circuits perpetuate both inflammation and CP. An overview of the key players and mechanisms can be visualized in **Figure 1**.

## 3 Established type 2–driven dermatologic diseases

### 3.1 Atopic dermatitis

AD is a hallmark type 2 dermatosis in which epidermal barrier dysfunction, epithelial alarmin release, and Th2 polarization reinforce one another, perpetuating chronic inflammation and pruritus. Damaged keratinocytes release TSLP, IL-25, and IL-33, activating ILC2s and Th2 cells and amplifying downstream IL-4, IL-13, and IL-31 signaling (31). Barrier dysfunction is further amplified by FLG loss-of-function variants and by direct IL-4/IL-13–mediated suppression of structural proteins essential for epidermal integrity, creating a chronic cycle of inflammation and impaired barrier repair (32, 33).

IL-4 and IL-13 are principal drivers of AD pathogenesis, promoting IgE production, eosinophilic inflammation, and chronic Th2 polarization (34). IL-31 is a major pruritogenic cytokine in AD (35, 36). Produced by Th2 cells, macrophages, basophils, eosinophils, and dendritic cells, IL-31 contributes substantially to itch and neuronal sensitization through signaling via IL-31RA/OSMRβ on keratinocytes and sensory nerves. IL-31 also functions within a broader neuroimmune itch network involving TSLP and periostin (28, 35, 36). Targeted biologics have further validated the central role of type 2 inflammation in AD. Dupilumab, a monoclonal antibody targeting IL-4Rα and thereby blocking both IL-4 and IL-13 signaling, produced substantial improvements in disease severity, pruritus, and quality of life in the SOLO 1 and SOLO 2 trials (37). Given its broad evidence base, young age approval, and ability to address atopic comorbidities, dupilumab often remains first-line in biologic treatment of AD (38). IL-13 inhibition with tralokinumab and lebrikizumab has also demonstrated significant clinical efficacy, making these reasonable alternatives in patients concerned about potential adverse effects of dupilumab (38–42). Nemolizumab, an IL-31RA antagonist, rapidly improves itch and epidermal differentiation in moderate-to-severe AD, further supporting the central role of IL-31 within the neuroimmune axis of AD (43).

### 3.2 Chronic spontaneous urticaria

CSU is a mast cell–predominant disorder characterized by recurrent wheals, angioedema, and intense pruritus (44–46). Mast cells, and to a lesser extent basophils, release histamine, leukotrienes, and pruritogenic cytokines that drive wheal formation, vascular permeability, and itch in CSU regardless of the underlying trigger (44–46). Autoimmune mechanisms underlying CSU involve either IgE or IgG autoantibodies (47), but both converge on enhanced FcϵRI signaling and spontaneous mast cell activation (48). Beyond histamine signaling, elevated IL-4, IL-5, and IL-13 levels have been identified in CSU patients, which together with tissue and peripheral eosinophilia, suggest that a subset of patients exhibit significant overlapping type 2 inflammatory features (49, 50).

These mechanistic insights have led to the use of targeted biologics in CSU, including omalizumab, which reduces free IgE levels and downregulates FcϵRI expression (51). High baseline IgE levels are a predictor of a faster, more robust response to omalizumab, whereas low baseline IgE levels predict a poorer response (52). Additionally, IL-4/IL-13 blockade with dupilumab has demonstrated efficacy in some patients with antihistamine-refractory CSU, including those with inadequate response to omalizumab, further supporting the concept that CSU may be partially driven by underlying type 2 inflammatory pathways (53–55). Importantly, dupilumab has since received FDA approval for CSU in patients aged 12 years and older who remain symptomatic despite H1 antihistamine therapy (56). In addition, growing evidence supports an important role for Bruton tyrosine kinase (BTK), a key intracellular signaling molecule downstream of FcϵRI that helps drive mast cell and basophil activation across both autoimmune CSU endotypes. This has led to the development of BTK inhibitors such as remibrutinib, an oral targeted therapy that demonstrated significant clinical benefit in the phase 3 REMIX-1 and REMIX-2 trials and has since received FDA approval for antihistamine-refractory CSU (57). Importantly, because BTK inhibition acts downstream of receptor activation, this strategy may help suppress mast cell activation regardless of whether disease is driven primarily by IgE autoantibodies or IgG-mediated autoimmune mechanisms (56, 57).

### 3.3 Prurigo nodularis

PN is a chronic pruritic disorder characterized by marked type 2 inflammation and neuroimmune dysregulation (58–60), with elevated IL-4, IL-13, IL-31 eosinophilic infiltration and periostin expression (58–62). Biomarker studies indicate substantial heterogeneity, suggesting that distinct inflammatory endotypes may underlie differences in clinical phenotype and treatment response (63). The success of targeted biologics has further established PN as a hallmark type 2 neuroimmune disorder. Dupilumab significantly improves both pruritus and nodule burden, supporting the importance of IL-4 and IL-13 in disease pathogenesis (9, 64). Similarly, nemolizumab produces rapid and often dramatic reductions in itch and sleep disturbance, reinforcing IL-31 as a central mediator of CP in PN (65, 66). This relationship was further highlighted in the pivotal phase 3 OLYMPIA 2 trial, in which 56.3% of patients treated with nemolizumab achieved at least a 4-point reduction in peak pruritus numerical rating scale scores at week 16 compared with 20.9% treated with placebo (65). Treatment was also associated with improvements in molecular markers of neuroimmune and epithelial dysregulation within lesional skin, providing biologic evidence of IL-31 involvement (65). Together, these therapies provide compelling clinical evidence for the intertwined immune and neural mechanisms underlying PN.

### 3.4 Bullous pemphigoid

BP is now understood as a type 2–amplified autoimmune blistering disorder rather than a purely autoantibody-mediated disease. Lesional biopsies and serum studies consistently demonstrate eosinophilic infiltration, elevated IL-4 and IL-13 levels, and increased IgE in BP (67, 68). In addition, severe pruritus often precedes blister formation and appears to share neuroimmune itch pathways with PN and other Th2-associated dermatoses (67, 68). Collectively, these findings suggest that neuroimmune and type 2 inflammatory pathways contribute substantially to symptom generation in BP in addition to autoantibody-mediated blistering.

Dupilumab use in BP cohorts and case series has demonstrated significant reductions in disease activity, blister burden, and pruritus, further supporting the role of IL-4/IL-13 signaling in disease propagation (69). More recent studies have substantially strengthened the evidence supporting dupilumab in BP. Multiple retrospective studies, a meta-analysis, and the phase 3 ADEPT trial demonstrated significant improvements in disease activity, pruritus, and corticosteroid-sparing outcomes with dupilumab, ultimately leading to FDA approval for BP and providing strong clinical validation for the role of type 2 inflammation in BP pathogenesis (70, 71). Likewise, omalizumab has shown benefit in selected patients by targeting IgE-mediated pathways, reinforcing the contribution of Type 2 humoral immunity to BP pathobiology (72, 73).

## 4 Emerging type 2–associated dermatoses

A growing number of conditions traditionally classified outside the atopic spectrum have demonstrated clinical and mechanistic responses to type 2–targeted therapies and appear to share overlapping Th2, eosinophilic, and neuroimmune features that may contribute to chronic inflammation and refractory pruritus.

### 4.1 Chronic pruritus of unknown origin

Chronic pruritus of unknown origin (CPUO) appears to include a Th2-predominant endotype characterized by elevated IL-31 and IL-33 (74). Clinical responses to dupilumab, nemolizumab, and abrocitinib suggest that a subset of patients harbor previously underrecognized inflammatory endotypes that may be amenable to targeted immunomodulatory therapy (9) (74, 75).

### 4.2 Eosinophilic dermatoses

Several eosinophilic dermatoses further highlight the expanding therapeutic relevance of type 2 immune targeting in dermatology. Wells syndrome is characterized by dense dermal eosinophilic infiltrates, flame figures, and elevated IL-5 signaling, and has demonstrated responses to anti-IL-5 and anti-IL-5Rα therapies including mepolizumab and benralizumab, as well as to IL-4/IL-13 blockade with dupilumab (76–79). Additional reports of response to IL-5-, IL-5Rα-, and IL-4/IL-13-targeted therapies in hypereosinophilic syndrome and eosinophilic annular erythema further support the contribution of type 2 pathways to eosinophilic skin disease (80) (81, 82).

### 4.3 Lichen simplex chronicus

Lichen simplex chronicus (LSC) is increasingly recognized as a neuroimmune disorder with significant type 2 inflammatory features (83). Although the itch–scratch cycle remains the central driver of disease chronicity (83, 84), repetitive mechanical trauma can itself promote type 2 immune activation through keratinocyte release of the epithelial alarmins TSLP, IL-33, and IL-25 (85). Histopathologic studies of chronically lichenified skin demonstrate inflammatory infiltrates containing T cells, macrophages, eosinophils, and IgE-bearing Langerhans cells, accompanied by increased expression of type 2 cytokines including IL-4, IL-5, and IL-13 (85, 86). Interestingly, LSC plaques have been found to have decreased density of cutaneous nerve fibers, suggesting subsequent development of abnormal sensitivities leading to pathological itch sensation and highlighting the complex interplay between neural and immune pathways (84). Consistent with these observations, emerging evidence supports the continued investigation of type 2-targeted therapies, including dupilumab, for treatment-refractory LSC.

## 5 Biologics as functional validation of type 2 pathways

The clinical efficacy of targeted biologic therapies provides strong evidence that type 2 immune pathways are central drivers of disease pathogenesis. Dupilumab, which blocks the IL-4Rα and thereby inhibits both IL-4 and IL-13 signaling, has demonstrated efficacy in AD (37), PN (64), CSU (53), and BP (70). Across these conditions, treatment produces substantial improvements in disease severity and pruritus while reducing reliance on systemic immunosuppression. More selective targeting of IL-13 with tralokinumab (87) and lebrikizumab (40) has similarly yielded significant clinical benefit in AD, confirming that IL-13 is a key downstream effector of type 2 inflammation. The efficacy of omalizumab, an anti-IgE monoclonal antibody, in CSU and treatment-refractory BP (88) further highlights the importance of IgE-mediated mast cell activation as a downstream consequence of type 2 immune signaling (89, 90). In contrast, therapies targeting eosinophils, including anti-IL-5 and anti-IL-5Rα antibodies, have shown largely disappointing results in dermatologic diseases despite their established efficacy in systemic eosinophilic disorders (91, 92). Importantly, blockade of IL-31 signaling with nemolizumab results in rapid and profound itch reduction in both AD and PN, providing direct evidence for the central role of neuroimmune pathways in CP (65). However, nemolizumab has less anti-inflammatory effect in AD than expected (93). Together, the success of biologics targeting IL-4/IL-13, IgE, and IL-31 signaling demonstrates that type 2 inflammation is not merely associated with these disorders but represents a fundamental pathogenic driver and therapeutically actionable target across diverse dermatologic diseases (**Table 1**).

**Table 1:** Overview biologics targeting mediators of type 2 inflammation in dermatologic conditions, including FDA-approved indications and emerging clinical applications.

Biologic	Target	FDA-approved dermatologic indication(s)	Emerging dermatologic indications under investigation
Dupilumab	IL-4Rα (blocks IL-4/IL-13 signaling)	Atopic dermatitis, prurigo nodularis, chronic spontaneous urticaria, bullous pemphigoid	Chronic pruritus of unknown origin (CPUO), lichen simplex chronicus (LSC), alopecia areata, chronic hand eczema (107)
Tralokinumab	IL-13	Atopic dermatitis	None
Lebrikizumab	IL-13	Atopic dermatitis	None
Nemolizumab	IL-31Rα	Prurigo nodularis, atopic dermatitis	CPUO, cholestatic pruritus, chronic kidney disease-associated pruritus (108, 109)
Omalizumab	IgE	Chronic spontaneous urticaria	Bullous pemphigoid (110)
Mepolizumab	IL-5	None	Bullous pemphigoid (111), hypereosinophilic syndrome with cutaneous involvement, eosinophilic dermatoses (112)
Benralizumab	IL-5Rα	None	Bullous pemphigoid (111), eosinophilic dermatoses

### 5.1 Safety concerns and paradoxical reactions

Despite their efficacy, some type 2–targeted therapies are associated with class- and agent-specific adverse effects that warrant consideration. Dupilumab is associated with conjunctivitis (37), as well as dupilumab-associated facial and head/neck erythema (94), both of unclear mechanism and occasionally treatment-limiting. Paradoxical inflammatory phenomena have also been reported, including new-onset or worsening psoriasiform dermatitis and alopecia areata during IL-4/IL-13 blockade, suggesting that suppression of type 2 signaling may unmask or potentiate Th1/Th17-skewed pathways in predisposed patients (95). Transient peripheral eosinophilia is common with IL-4Rα and IL-13 blockade and, though usually asymptomatic, has rarely been associated with eosinophilic complications (96). Omalizumab carries a boxed warning for anaphylaxis, necessitating post-injection observation (97). Bruton tyrosine kinase inhibitors such as remibrutinib carry class-associated risks including bleeding tendency, however this is generally mild (98). Taken together, these findings emphasize that type 2–targeted therapies are not uniformly benign and that patient selection, monitoring, and shared decision-making remain essential components of care.

## 6 Future directions

Future advances in type 2 dermatology will likely arise from improved biomarker identification, refinement of disease endotypes, and deeper understanding of neuroimmune signaling pathways. Emerging data suggest that AD can be divided into distinct inflammatory subtypes, including Th2-dominant, Th2/Th22, and Th2/Th17 phenotypes, which may help explain differences in response to targeted therapies and facilitate precision medicine approaches (99–101). Increasing evidence also suggests that these inflammatory endotypes vary according to race, ethnicity, age, and disease chronicity (10). Chronic AD lesions have additionally been shown to develop increased Th1 infiltration while maintaining persistent Th2 and Th22 activity, highlighting the dynamic and evolving nature of disease-associated immune signaling (10). Recent *in vitro* studies further demonstrated that the combination of IL-22 with Th2 cytokines most closely reproduces key epidermal features of AD, suggesting that the Th2/Th22 endotype may play a particularly important role in driving epidermal pathology (102). Similar approaches are needed in CSU, PN, BP, and eosinophilic dermatoses to better identify distinct inflammatory subtypes and guide treatment (99–101).

Further characterization of neuroimmune pathways, including the cytokines, neuropeptides, ion channels, and sensory neurons involved in CP will be important for development of future targeted therapies (103, 104). This is particularly relevant because itch severity often does not correlate well with the degree of visible inflammation, suggesting that chronic neuronal sensitization may persist even after inflammation improves.

In addition, tissue-resident memory T cells may persist within clinically resolved skin and contribute to recurrent disease activity and relapse, even in the absence of overt inflammation (105). This concept may help explain why some patients rapidly flare after treatment discontinuation and has generated interest in therapies targeting the OX40/OX40L signaling pathway as a potential strategy for longer-term disease modification rather than temporary inflammatory suppression alone (105).

Recent approvals of therapies targeting IL-4Rα, IgE, and IL-31 signaling have expanded treatment options across numerous inflammatory and pruritic skin disorders (106). Future integration of biomarker profiling with clinical phenotyping may enable more personalized therapeutic selection, improve long-term outcomes, and broaden the application of type 2-targeted therapies throughout dermatology.

## 7 Conclusion

Type 2 inflammation has emerged as a unifying immunologic framework underlying a growing spectrum of dermatologic diseases, including AD, PN, CSU, and BP. Advances in our understanding of epithelial barrier dysfunction, alarmin signaling, immune cell activation, and neuroimmune crosstalk have revealed complex interactions between keratinocytes, immune cells, fibroblasts, and sensory neurons that drive chronic inflammation and pruritus. The remarkable clinical success of biologic therapies targeting IL-4/IL-13, IgE, and IL-31 pathways has both transformed patient care and provided powerful validation of the central role of type 2 immunity in cutaneous disease. However, differential responses to therapies targeting distinct components of the type 2 pathway highlight the heterogeneity and complexity of these disorders. Continued investigation into epithelial, immune, stromal, and neuronal interactions will further refine disease endotypes, enable more personalized therapeutic strategies, and expand the application of type 2-targeted therapies across dermatology.
